# MicroRNA-130b targets PTEN to mediate drug resistance and proliferation of breast cancer cells via the PI3K/Akt signaling pathway

**DOI:** 10.1038/srep41942

**Published:** 2017-02-06

**Authors:** Yuan Miao, Wei Zheng, Nana Li, Zhen Su, Lifen Zhao, Huimin Zhou, Li Jia

**Affiliations:** 1 College of Laboratory Medicine, Dalian Medical University, Dalian 116044, Liaoning Province, China; 2 Department of Central Laboratory, the First Affiliated Hospital of Dalian Medical University, Dalian 116011, Liaoning Province, China; 3 Graduate School, Dalian Medical University, Dalian 116044, Liaoning Province, China; 4 Department of Microbiology, Dalian Medical University, Dalian 116044, Liaoning Province, China

## Abstract

Multidrug resistance (MDR) correlates with treatment failure and poor prognosis among breast cancer patients. This study was aimed to investigate the possible mechanism by which microRNA-130b-3p (miR-130b) mediates the chemoresistance and proliferation of breast cancer. MiR-130b was found to be up-regulated in tumor tissues versus adjacent tissues of breast cancer, as well as in adriamycin (ADR) resistant breast cancer cell line (MCF-7/ADR) versus its parental line (MCF-7) and the non-malignant breast epithelial cell line (MCF-10A), demonstrating its crucial relevance for breast cancer biology. We identified that PTEN was a direct target of miR-130b and inversely correlated with miR-130b expression in breast cancer. Moreover, over-expression of miR-130b promoted drug resistance, proliferation and decreased apoptosis of MCF-7 cells, while suppression of miR-130b enhanced drug cytotoxicity and apoptosis, as well as reduced proliferation of MCF-7/ADR cells *in vitro* and *in vivo.* Particularly, miR-130b mediated the activity of phosphoinositide-3 kinase (PI3K)/Akt signaling pathway as well as the chemoresistance and proliferation of breast cancer cell lines, which was partially blocked following knockdown of PTEN. Altogether, miR-130b targets PTEN to induce MDR, proliferation, and apoptosis via PI3K/Akt signaling pathway. This provides a novel promising candidate for breast cancer therapy.

Breast cancer (BC) is one of the most universal malignant tumors of worldwide women and is a significant health problem in terms of both morbidity and mortality. About 178,480 new cases of invasive BC were diagnosed in 2007, and 40,460 women will die of this cancer in USA^1^. The main treatment strategies are the combination of surgery and adjuvant therapy, for instance, anticancer drugs, hormonal therapy, targeted drugs or a combination thereof^2^. However, the major barrier to successful treatment is multiple drug resistance in BC. It is clearly suggested that the drug resistance was a major obstacle to successful treatment in BC patients^2^ and increasing attention has been paid to the effects of miRNAs on the development of cancer drug resistance recently^3^,^4^,^5^,^6^.

MicroRNAs (miRNAs) are small non-coding RNAs (20–25 nucleotides) that result in a downregulation of target proteins through the degradation of this mRNA or through translational inhibition^7^, which play an important role in various malignancies. Aberrant expression of miRNAs has been reported to participate in physiological and pathological processes of a variety of human cancers, such as proliferation^8^, invasion^9^, apoptosis^10^ and chemotherapy resistance^11^. MiR-130b targets CYLD to inhibit proliferation and induce apoptosis in human gastric cancer cells^12^. MiR-130b targets PTEN to promote children APL progression by promoting cell proliferation and inhibiting apoptosis^13^. Moreover, it has been reported that miR-130b was up-regulated in triple-negative BC compared with adjacent normal tissue and miR-130b-5p mediated CCNG2 that may be related to the malignant progression of triple-negative BC^14^.

PTEN is one of the most commonly tumor suppressor gene in human cancers and takes an important role in the regulation of cell growth and apoptosis^15^. PTEN has been reported to be targeted by many miRNAs. MiRNA-21 induces epithelial to mesenchymal transition and gemcitabine resistance via the PTEN/AKT pathway in BC^16^. MiR-221 reduces the sensitivity of cervical cancer cells to gefitinib through the PTEN/PI3K/Akt signaling pathway^17^. MiR-106b induces cell radioresistance via the PTEN/PI3K/AKT pathway in colorectal cancer^18^. But the biological role of miR-130b in modulating the breast cancer drug resistance and proliferation by targeting PTEN through PI3K/Akt signaling pathway has been unexplored.

In the present study, we investigated the expression levels of miR-130b and PTEN in tumor and adjacent tissues of BC patients and in the parental and chemo-resistant BC cell lines, in order to identify the functional role of miR-130b in BC biology. Moreover, we elucidated the regulatory PI3K/Akt pathway involving miR-130b and PTEN in BC cell multidrug resistance and proliferation development.

## Results

### Expression level of miR-130b in BC tissues and cell lines

**Figure 1:**
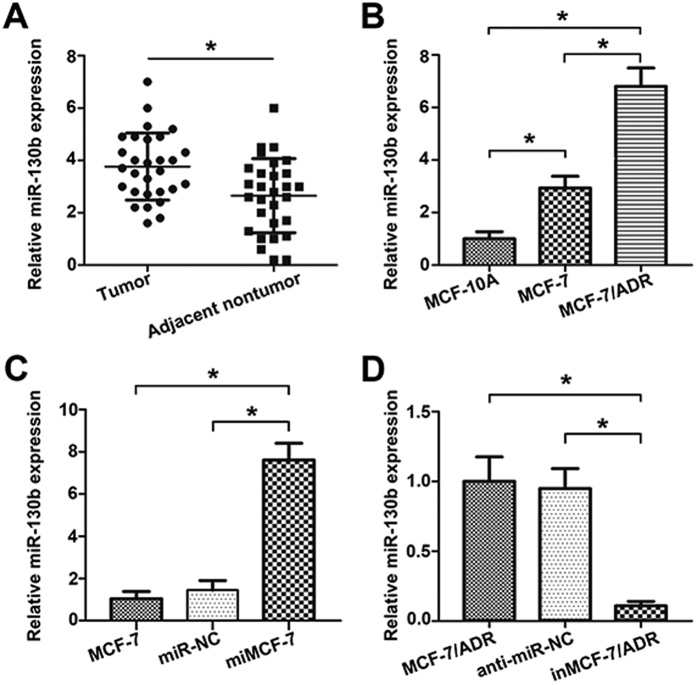
Relative miR-130b levels in BC tissues and cells were detected by real-time RT-PCR. (**A**) MiR-130b expression level was up-regulated in BC samples (n = 29) relative to matched adjacent normal breast tissue (n = 29). (**B**) MiR-130b expression in MCF-7/ADR cells was the highest among these three cell lines, and the expression of miR-130b in MCF-7 cells was higher than in MCF-10A cells. (**C**,**D**) Differential expression of miR-130b was shown in MCF-7, MCF-7/ADR cells and their transfected cells (*P < 0.05). The results were showed as mean ± SD of three independent assays.

To study the role of miR-130b in BC cells, firstly, 29 samples of patients with BC were detected in this study, as shown in Fig. 1A, the expression of miR-130b was significantly up-regulated in BC samples compared to matched adjacent normal breast tissue. Furthermore, we measured miR-130b expression levels in BC cell lines by quantitative real-time PCR (qRT-PCR). As shown in Fig. 1B, the expressions of miR-130b was found to be up-regulated in MCF-7 and MCF-7/ADR cells in contrast to the expression level of non-malignant breast epithelial cell line, MCF-10A. Additionally, compared with MCF-7 cell line, miR-130b was highly expressed in MCF-7/ADR cell line. Over-expression of miR-130b in MCF-7 cells (miMCF-7) and depletion of miR-130b in MCF-7/ADR (inMCF-7/ADR) were built by transfecting with miR-130b mimics or miR-130b inhibitor, respectively (Fig. 1C and D).

### PTEN is a direct target gene of miR-130b and inversely correlates with miR-130b expression in BC

**Figure 2:**
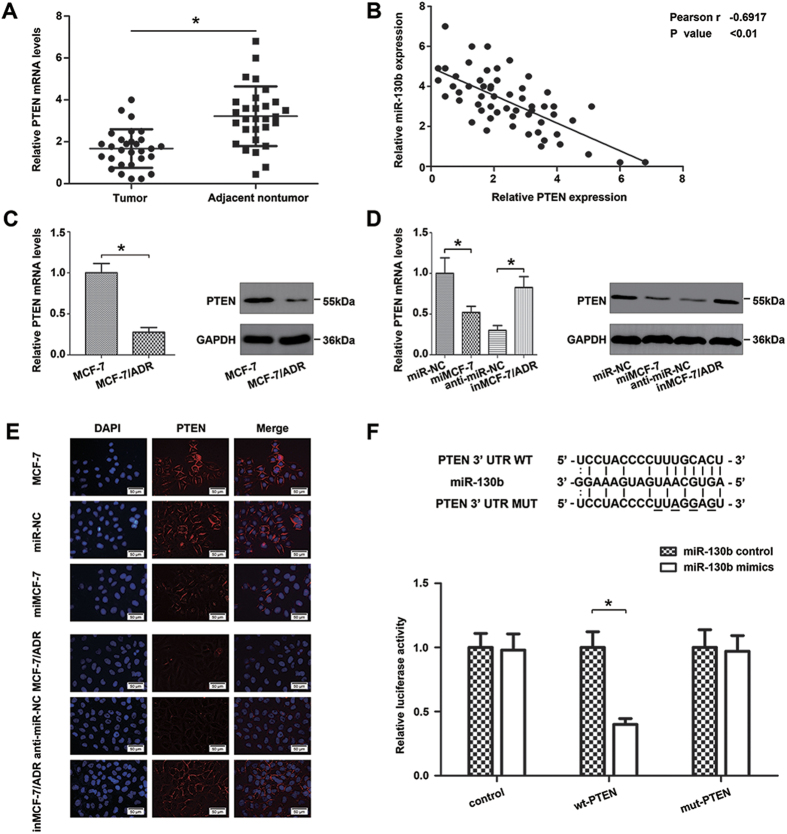
PTEN is a direct target gene of miR-130b. (**A**) PTEN was detected by qRT- PCR in 29 BC tissues and was matched the adjacent noncancerous breast tissues. (**B**) The relationship between PTEN and miR-130b expression was explored by Spearman’s correlation in 29 paired BC tissues and adjacent noncancerous breast tissues. PTEN was inversely correlated with the miR-130b levels in BC tissues. (**C**) Relative PTEN expression in MCF-7 and MCF-7/ADR cells were detected by qRT-PCR (left panel) and western blot (right panel). (**D**) Effects of miR-130b mimics in MCF-7 cells and miR-130b inhibitor in MCF-7/ADR cells on the relative expression levels of PTEN were detected by qRT- PCR (left panel) and western blot (right panel). (**E**) PTEN expression was detected by immunofluorescence staining in these cells. Red fluorescence: PTEN, blue fluorescence: DAPI, staining for nuclear DNA. There were no significant differences between the control group and the blank group. (**F**) The relative luciferase activity of 293 T cells was detected after the pGL3-control vector, wt or mut PTEN 3′UTR genes were co-transfected with miR-NC or miR-130b mimics (*P < 0.05). All data are mean ± SD of three independent assays.

The differences in expression of PTEN between BC tissues and adjacent normal tissues were determined using qRT-PCR. As shown in Fig. 2A, the expression level of PTEN in BC adjacent nontumor tissues was significantly higher than in the BC tissues. Moreover, there was an inverse correlation between the expression of miR-130b and PTEN (Fig. 2B). qRT - PCR and western blot were also used to determine the differential expression of PTEN between MCF-7 cells and MCF-7/ADR cells. Figure 2C showed that reduced expression of PTEN at both mRNA and protein levels (*P < 0.05) in MCF-7/ADR cells compared to MCF-7 cells. Then we sought to verify whether miR-130b exerts its regulatory function by targeting PTEN, miR-NC, miMCF-7, anti-miR-NC and inMCF-7/ADR cells were examined for PTEN expression by qRT-PCR, western blot and immunofluorescence staining assay. As shown in Fig. 2D and E, up-regulated miR-130b led to an obvious decrease in PTEN expression. Reversely, suppressing miR-130b resulted in an up-regulation of PTEN expression.

To assess whether miR-130b can directly alter the expression of PTEN in BC cells, wild-type and mutant 3′-UTR PTEN target sequences were cloned into the pGL3 luciferase reporter vector (Fig. 2F), a dual-luciferase reporter assay indicated that the co-transfection of miR-130b mimics and the PTEN-3′-UTR-wild vector into 293 T cells conspicuously repressed the luciferase activity compared to the co-transfection of control vectors and miR-130b mimics. However, the suppression of luciferase activity was blocked by co-transfection of miR-130b mimics and the 3′-UTR mutant of PTEN. In addition, MCF-7 cells were co-transfected with PTEN 3′-UTR luciferase reporter construct and miR-130b mimics or NC mimic. The result was shown in Supplemental Fig. 1. As expected, miR-130b inhibited the luciferase expression of the PTEN WT 3′UTR construct, which was de-repressed by mutating the miR-130b seed sequence within the PTEN 3′-UTR. The results showed the same tendency in 293 T cells. These data suggest that PTEN is a direct target of miR-130b. MiR-130b inversely accommodates PTEN expression by directly interacting with their 3′-UTR.

### Over-expression of miR-130b induces MCF-7 cells resistance to chemotherapy and promotes proliferation *in vitro* and *in vivo*

**Figure 3:**
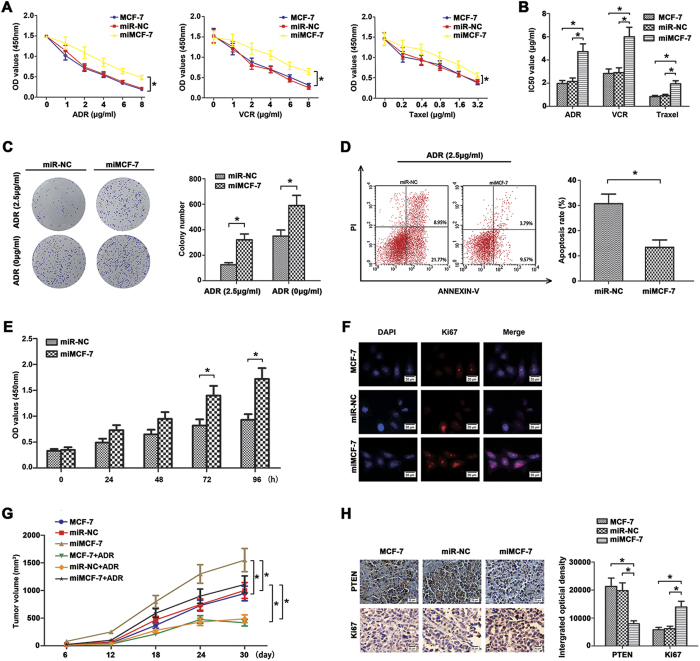
Over-expression miR-130b enhanced chemoresistance and proliferation of MCF-7 cells *in vitro* and *in vivo*. (**A**) After transfection, cells were treated with different concentrations of ADR, VCR and Taxel for 48 h, respectively. Cell absorbances (450 nm) were assessed by CCK8 assay. (**B**) The reported values were the IC50 (mean ± SD) of three independent experiments. IC50 represents the drug concentration producing 50% decrease of cell growth. There were no significant differences between the control group and the blank group. (*P < 0.05; miR-130b mimics vs miR-NC). (**C**) Effects of miR-130b on the formation of cell clones when cells were treated with or without ADR (2.5 μg/ml) were analyzed by clone formation assay. Transfection of the MCF-7 cells with miR-130b mimics decreased their sensitivity to ADR treatment and enhanced their clone formation ability. (**D**) Decreased apoptosis rate were measured by flow cytometry assay. (**E**) The viabilities of MCF-7 cells transfected with miR-130b mimics or miR-NC were detected by CCK8 assay at 0, 24, 48, 72 and 96 h. (**F**) Ki67 expression was detected by immunofluorescence staining in these cells. Red fluorescence: Ki67, blue fluorescence: DAPI, staining for nuclear DNA. There were no significant differences between the control group and the blank group. (**G**) The miR-130b enhanced the tumourigenicity in the nude mice xenograft model. The tumor growth curves at 30 days are shown as indicated. (**H**) Deregulation of PTEN (×400) and Ki67 (×400) expressions were showed using IHC staining in xenograft tumors derived from MCF-7, miR-NC and miMCF-7 cells. There were no significant differences between the control group and the blank group (*P < 0.05). The results were showed as mean ± SD of three independent experiments.

To identify the role of miR-130b in MCF-7 cell chemoresistance *in vitro*, CCK8 assays indicated that miR-130b over-expression significantly enhanced the cell viability to various chemotherapeutics (ADR, VCR, Taxel) (Fig. 3A, *P < 0.05). In addition, changes of IC50 values for these chemotherapeutic agents were found in these transfected cells (Fig. 3B, *P < 0.05). The colony formation assay was used to explore the effect of overexpressed miR-130b on the colony formation rate in the presence of ADR (2.5 μg/ml). As shown in Fig. 3C, over-expression of miR-130b resulted in an increased colony formation rate to ADR compared with their control cells (*P < 0.05). Then we examined whether miR-130b was capable of inhibiting ADR-induced apoptosis in MCF-7 cells. As shown in Fig. 3D, flow cytometry assay revealed markedly lower apoptotic rate in miMCF-7 cells than negative control cells (*P < 0.05).

The role of miR-130b in MCF-7 cell proliferation *in vitro* was assessed using CCK8, colony formation and immunofluorescence assays. The CCK8 absorbances were measured at 0, 24, 48, 72 and 96 h, Fig. 3E clearly shown that increased growth rate of miMCF-7 compared with miR-NC cells (*P < 0.05). Colony formation assay showed significantly more colonies in miMCF-7 cells than in miR-NC cells (Fig. 3C). Numerous studies have reported the appreciation value of Ki67 in BC^19^,^20^. Compared to the control groups, immunofluorescence staining assay demonstrated that over-expression of miR-130b could increase the expression of Ki67 (Fig. 3F, *P < 0.05). These results indicated that miR-130b facilitated proliferation of MCF-7 cells *in vitro*, which is in agreement with other studies displaying analogous results on hepatocellular carcinoma^21^.

To further confirm the above findings, BALB/c nude mice were injected with MCF-7, miR-NC, miMCF-7 cells (2 × 10^6^ cells per mouse) in the right flank respectively. After 6 days, the mice were randomly assigned to groups (n = 6/group) and exposed to ADR (7 mg/kg) or 0.9% physiological saline as control weekly. Figure 3G showed that tumor volume of MCF-7 combined with ADR was significantly smaller than in miMCF-7 group at the end of the experiment. Moreover, throughout the tumorigenic period, tumors from the over-expression miR-130b cells grew significantly faster than their negative controls (Fig. 3G). Furthermore, immunohistochemistry staining assay showed lower expression of PTEN and higher expression of Ki67 in miR-130b overexperssion groups than in the control groups (Fig. 3H). Taken together, *in vitro* and *in vivo* studies confirmed that over-expression of miR-130b promotes ADR resistance and proliferation in MCF-7 cells.

### Down-regulation of miR-130b induces cells chemosensitivity and inhibits proliferation of MCF-7/ADR cells *in vitro* and *in vivo*

**Figure 4:**
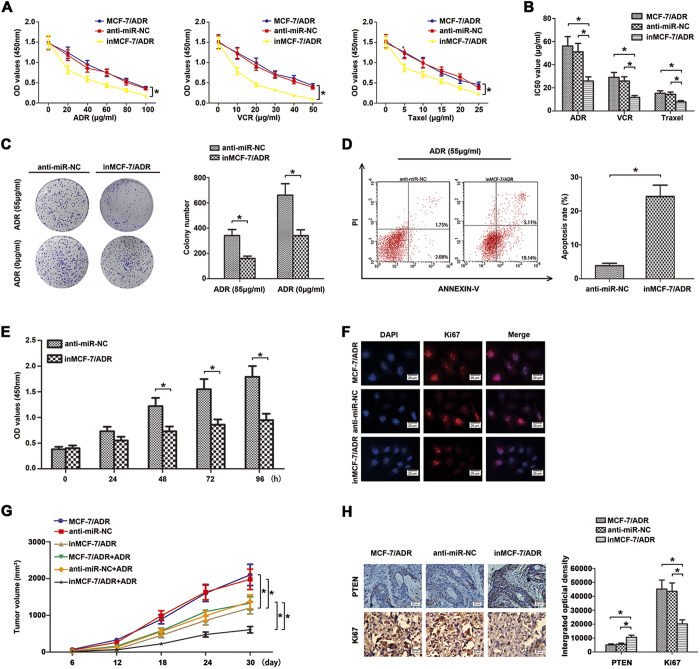
Down-regulation of miR-130b enhanced chemosensitivity and reduced proliferation of MCF-7/ADR cells *in vitro* and *in vivo*. (**A**) After transfection, cells were treated with different concentrations of ADR, VCR and Taxel for 48 h, respectively. Cell absorbances (450 nm) were measured by CCK8 assay. (**B**) The reported values were the IC50 (mean ± SD) of three independent experiments. Decreased IC50 values resulted from transfecting with miR-130b inhibitor of MCF-7/ADR cells, but there were no significant differences between the control group and the blank group (*P < 0.05; miR-130b inhibitor vs anti-miR-NC). (**C**) Effects of down-regulated miR-130b on the formation of cell clones when cells were treated with or without ADR (55 μg/ml) were analyzed by clone formation assay. Transfection of the MCF-7/ADR cells with miR-130b inhibitor increases their sensitivity to ADR treatment and reduces their proliferation ability. (**D**) MCF-7/ADR cells transfected with miR-130b inhibitor resulted in increased apoptosis rate compared with anti-miR-NC. (**E**) The bar graph of proliferation of inMCF-7/ADR cells compared to control cells were detected by CCK8 assay at 0, 24, 48, 72 and 96 h. (**F**) Ki67 expression was detected by immunofluorescence staining in these cells. Red fluorescence: Ki67, blue fluorescence: DAPI, staining for nuclear DNA. There were no significant differences between the control group and the blank group. (**G**) Down-regulation of miR-130b reduced the tumourigenicity in the nude mice xenograft model. The tumor growth curves at 30 days are shown as indicated. (**H**) Enhanced expression of PTEN and decreased expression of Ki67 were shown using IHC staining in xenograft tumors derived from MCF-7/ADR, anti-miR-NC and inMCF-7/ADR cells. There were no significant differences between the control group and the blank group (*P < 0.05). The results are showed as mean ± SD of three independent expriments.

To identify the role of miR-130b in MCF-7/ADR cell chemoresistance *in vitro*, CCK8 assays indicated that miR-130b down-regulation significantly decreased the cell viability to various chemotherapeutics (Fig. 4A, *P < 0.05). In addition, changes of IC50 values for these chemotherapeutic agents were found in these transfected cells (Fig. 4B, *P < 0.05). The colony formation assay showed that downregulated miR-130b resulted in a decreased colony formation rate to ADR (55 μg/ml) than their control cells (Fig. 4C,*P < 0.05). As shown in Fig. 4D, flow cytometry assay revealed markedly higher apoptotic rate in inMCF-7/ADR cells than negative control cells (*P < 0.05).

CCK8, colony formation and immunofluorescence assays were used to testify the role of miR-130b supression in MCF-7/ADR cell proliferation *in vitro*. CCK8 absorbances clearly showed that decreased growth rate of inMCF-7/ADR compared with anti-miR-NC cells measured at 0, 24, 48, 72 and 96 h (Fig. 4E, *P < 0.05). Colony formation assay confirmed apparently less colonies in inMCF-7/ADR cells than in anti-miR-NC cells (Fig. 4C, *P < 0.05). Immunofluorescence staining assay verified that suppression of miR-130b could reduce the expression of Ki67 compared to the control cells (Fig. 4F, *P < 0.05).

To strengthen the above functional evidence of miR-130b in MCF-7/ADR cells chemoresistance and proliferation, BALB/c nude mice were injected with MCF-7/ADR, anti-miR-NC, inMCF-7/ADR cells (2 × 10^6^ cells per mouse) in the right flank respectively. 6 days later, the mice were randomly assigned to groups (n = 6/group) and exposed to ADR (7 mg/kg) or 0.9% physiological saline as control weekly. Figure 4G showed a significant reduction of mean tumor volume of inMCF-7/ADR tumor, compared with that of the control group and the parental MCF-7/ADR group. Moreover, miR-130b inhibitor slowed the growth of tumor volume in inMCF-7/ADR group compared with MCF-7/ADR and anti-miR-NC groups. Furthermore, increased expression levels of PTEN and reduced expression levels of Ki67 protein in tumor cells inMCF-7/ADR were also approved by immunohistochemistry staining assay (Fig. 4H). These results demonstrated that down-regulation of miR-130b inhibited ADR resistance and proliferation in MCF-7/ADR cells *in vitro* and *in vivo*.

### Over-expression of miR-130b stimulates drug-resistance and proliferation via the PTEN-PI3K/Akt pathway in MCF-7 cells

**Figure 5:**
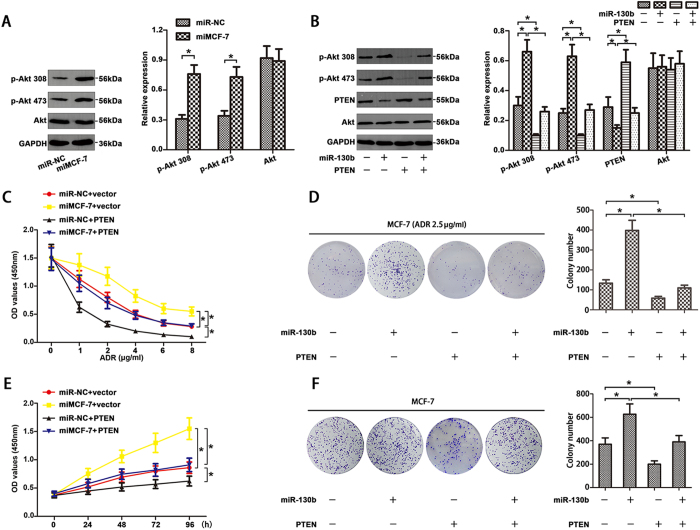
Over-expression miR-130b enhanced chemoresistance and proliferation by PTEN-PI3K/Akt pathway in MCF-7 cells. (**A**) MiR-130b mimics increased the expression of p-Akt 308 and p-Akt 473 in MCF-7 cells detected by western blot. (**B**) MCF-7 cells were co-transfected with PTEN pEGFP-N2 vector/control vector and miRNA mimics/miR-NC. Increased p-Akt 308 and p-Akt 473 levels and decreased PTEN expression were not discovered in MCF-7 cells transfected with miR-130b mimics + PTEN and total amount of Akt expression was not affected by miR-130b transfection. Up-regulation of PTEN blocked the effects induced by miR-130b over-expression in MCF-7 cells. Representative results were CCK-8 assay (**C**,**E**) and colony formation assay (**D**,**F**) and cells were treated with or without 2.5 μg/ml ADR (*P < 0.05). The results are showed as mean ± SD of three independent expriments.

To confirm whether miR-130b affects the PI3K/Akt signaling pathway in MCF-7 cells, western blot were carried out to analysis the protein expression levels of PI3K/Akt pathway. Figure 5A showed that protein levels of phospho-Akt 308 (p-Akt 308) and phospho-Akt 473 (p-Akt 473) expression were enhanced in miMCF-7 cells compared to the miR-NC. But total Akt expression level was not changed. Our results confirmed that miR-130b is a potential regulator of PI3K/Akt signaling pathway in BC cells.

It has been reported that PTEN acted as a tumor inhibitor gene by specifically reversely regulating the PI3K/Akt pathway^22^. Then we next explore whether miR-130b regulated Akt phosphorylation particularly by down-regulating PTEN, miR-NC + vector (It means that the cells were transfected with miR-NC and PTEN control vector at the same time. The following statements are identical), miR-130b mimics + vector, miR-NC + PTEN or miR-130b mimics + PTEN were co-transfected into MCF-7 cells respectively and the expression of p-Akt 308, p-Akt 473, Akt and PTEN were analyzed by western blotting. As showed in Fig. 5B, increased p-Akt 308 and p-Akt 473 expression and decreased PTEN expression were not discovered in MCF-7 cells transfected with miR-130b mimics + PTEN and total amount of Akt expression was not affected by miR-130b expression. These results demonstrated that miR-130b may activate PI3K/Akt signaling by silencing PTEN.

In MCF-7 cells, we treated the cells with a series of concentration gradients of ADR (0, 1, 2, 4, 6, 8 μg/ml). CCK8 assays (Fig. 5C) showed that miR-130b induced drug resistance was reversed by the restoration of PTEN expression (*P < 0.05). Similar results were found in colony formation assays when these cells were treated with ADR (2.5 μg/ml) (Fig. 5D). For proliferation, CCK8 (Fig. 5E) and colony formation (Fig. 5F) assays showed that increased proliferation rate and the amount of colonies were reversed by cells transfecting with miR-130b mimics + PTEN compared to miR-130b mimics + vector. Taking together, the PTEN-PI3K/Akt pathway plays a considerable role in miR-130b mediated drug resistance and proliferation in BC.

### Down-regulation of miR-130b decreased drug-resistance and proliferation via the PTEN-PI3K/Akt pathway in MCF-7/ADR cells

**Figure 6:**
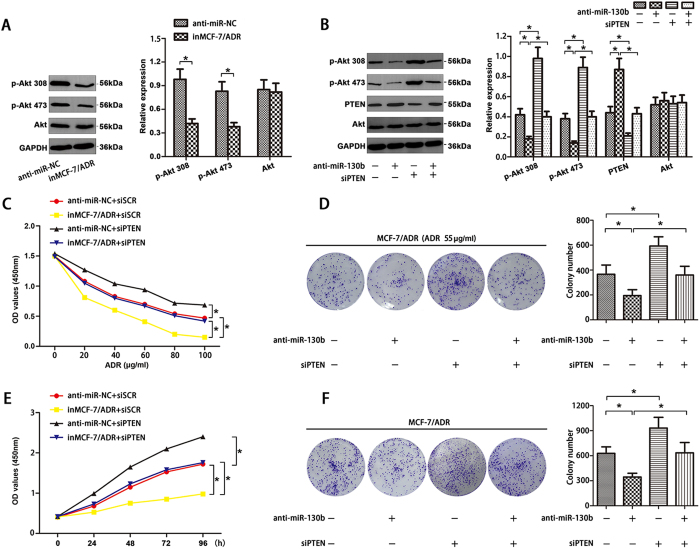
Down-regulation of miR-130b reduced chemoresistance and proliferation by PTEN/PI3K/Akt pathway in MCF-7/ADR cells. (**A**) MiR-130b inhibitor decreased the levels of p-Akt 308 and p-Akt 473 in MCF-7/ADR cells detected by western blot. (**B**) MCF-7/ADR cells were co-transfected with target mRNA (siPTEN or siSCR) and miRNA inhibitor (anti-miR-130b or anti-miR-NC). Decreased p-Akt 308 and p-Akt 473 levels and increased PTEN expression were not discovered in MCF-7/ADR cells co-transfected with anti-miR-130b + siPTEN and total amount of Akt expression was not affected by miR-130b transfection. Down-regulation of PTEN blocked the effects induced by miR-130b supression in MCF-7/ADR cells. Representative results were CCK-8 assay (**C**,**E**) and colony formation assay (**D**,**F**) and cells were treated with or without 55 μg/ml ADR (*P < 0.05). All data are mean ± SD of three independent assays.

Western blot were carried out to analysis the protein expression levels of PI3K/Akt pathway in anti-miR-NC and inMCF-7/ADR cells, respectively. Reduced expression of p-Akt 308 and p-Akt 473 was reviewed in inMCF-7/ADR cells (Fig. 6A). But total Akt expression level was not changed. Furthermore, co-transfected with miR-130b inhibitor + siPTEN in MCF-7/ADR cells did not induce the obvious changes of p-Akt 308, p-Akt 473 and PTEN expression (Fig. 6B) and Akt expression was not affected by miR-130b supression. To confirm whether miR-130b enhanced ADR resistance and proliferation specifically through the PI3K/Akt pathway in MCF-7/ADR cells, CCK-8 and colony formation assays showed that siRNA PTEN reversed the decreased chemoresistance (Fig. 6C and D, *P < 0.05) and proliferation (Fig. 6E and F, *P < 0.05) induced by transfecting with miRNA-130b inhibitor.

## Discussion

Evidence of miRNA-mediated drug resistance in malignant tumors is accumulating. There was a certain relationship between the deregulation of miRNAs and BC cells treating with chemotherapy. In this paper, we showed that the expression of miR-130b was significantly increased in BC tissue from patients and ADR-resistant BC cells (MCF-7/ADR cells).

MiRNAs, one of the most epidemic regulatory factors, for instance, miR-451^3^ and miR-224-3p^23^, are involved in the drug resistance to chemotherapeutic drug doxorubicin via regulating different targets in BC. Studies have also reported the associations of miR-130b with some types of solid tumors on multidrug resistance or proliferation. For multidrug resistance, miR-130a and miR-130b target NRP1 to mediate multidrug resistance in epithelial ovarian cancer^24^. Another study showed that, miR-130b promotes the development of drug sensitivity partially by targeting the 3′-UTR of CSF-1 in ovarian cancer^25^. For proliferation, miR-130b targets CYLD to inhibit proliferation and induces apoptosis in human gastric cancer cells^12^. Moreover, miR-130b inhibits cell proliferation and invasion in pancreatic cancer through targeting STAT3^26^. In contrast, over-expression miR-130b promotes the ability of proliferation in esophageal squamous cell carcinoma cells^27^ and in Hepatocellular carcinoma cells^21^. However, no published study has focused on the chemoresistance and proliferation mediated by miR-130b in BC. In this study, we found that miR-130b was overexpressed in BC tissues and drug resistance MCF-7/ADR cells. MiR-130b may be a potential regulator of BC resistance to chemotherapy and proliferation.

Past literature reported that the effect of miR-130b in colorectal cancer are due to PPARγ suppression that in turn deregulates PTEN, E-cadherin and so on^28^. It was also shown that disruption of the normal activity of the PTEN phosphatase increased BC chemotherapeutic drug resistance^29^. It has been reported that miRNA-17-5p promotes chemotherapeutic drug resistance and tumor metastasis by repressing PTEN expression in colon cancer cells^5^. We proved that PTEN expression was higher in the drug-sensitive cell lines than in the resistant cells. Altered expression of miR-130b also affects PTEN expression level. Over-expression of miR-130b in MCF-7 cells significantly inhibited PTEN expression and suppression of miR-130b in MCF-7/ADR cells increased PTEN expression apparently. Furthermore, taking advantage of bioinformatics research (Targetscan and MicroRNA.org assays) and dual-luciferase reporter assay, we found that PTEN, as a candidate of miR-130b targets, which were consistent with the previous study that PTEN is a direct target of miR-130b^27^. These results confirmed that PTEN involved in BC chemoresistance, proliferation and cancer development, and was negatively regulated by miR-130b.

Numerous studies have reported the relationship between miRNAs and chemoresistance^23^,^30^,^31^ or proliferation^32^. However, miR-130b whether plays a specific role in the drug resistance and proliferation of BC is surprisingly much less investigated. In our study, the manipulation of miR-130b expression modulated the BC cell drug resistance and proliferation *in vitro*. Further experiments demonstrated the regulated levels of miR-130b were correlated with BC cell progression *in vivo*. Up-regulation of miR-130b reduced the drug sensibility of BC cells to ADR, and enhanced tumor growth. Meanwhile, knockdown of miR-130b decreased chemoresistance and tumor growth. These results proved that miR-130b played an oncogenic role in BC cells as over-expression of miR-130b promotes cell ADR resistance and proliferation whereas the inhibiting miR-130b using anti-miR-130b suppresses BC cell ADR resistance and proliferation.

PTEN is a tumor-suppressing gene and negatively regulates the PI3K/Akt signalling pathway. Functionally, at the upstream of Akt, PTEN as a phosphatase to block the formation of phosphatidylinositol-3, 4, 5-trisphosphate (PIP3) from phosphatidylinositol-4, 5-bisphosphate (PIP2), thereby directly antagonized the activity of PI3 kinase (PI3K)^33^. Several studies have reported the oncogenic function of miR-130b regulated tumor development, progression and prognosis by directly targeting PTEN-PI3K/Akt signaling pathway. Our results provide further evidence that dysregulated expression of miR-130b mediated ADR resistance and proliferation specifically through the PTEN-PI3K/Akt pathway in MCF-7 and MCF-7/ADR cells, respectively. MiR-130b could positively regulate p-Akt308 and p-Akt473 expression in BC cells. Restoring the expression of PTEN in miR-130b overexpressed cells rescued the effect of miR-130b on p-Akt308 and p-Akt473 expression. Conversely, inhibition the expression of PTEN in miR-130b down-regulated cells restored the expression of p-Akt308 and p-Akt473. Most importantly, we focused on the relationship between deregulation of miR-130b and PTEN-PI3K/Akt on cells drug resistance and proliferation in BC. MiR-130b regulated the drug resistance and proliferation of BC cell lines by targeting PTEN and causing subsequent changes in the PI3K/Akt pathway, whereas negative regulation of PTEN by miR-130b was proved to counteract the positive influence on the drug resistance and proliferation via PI3K/Akt pathway, in other words, these abilities were impaired when cells were co-transfected with PTEN or siPTEN, respectively. These results demonstrated that the PTEN-PI3K/Akt pathway may be a potential mechanism through which miR-130b regulates the chemoresistance and proliferation development of BC.

In conclusion, miR-130b could induce chemoresistance and promote proliferation in BC cells by targeting PTEN through PI3K/Akt signaling pathway *in vitro* and *in vivo*. Recovery of PTEN expression could reverse miR-130b-induced cells chemoresistance and proliferation. These findings may provide important information for further experimental investigation on the mechanism of MDR-associated function and present potentially diagnostic or therapeutic strategies for BC.

## Materials and Methods

### Tissue Samples of Patients

Investigation was carried out in accordance with approved international guidelines and ethical standards. 29 pairs of BC tissues and their adjacent noncancerous tissues were collected from patients who underwent surgical resections at the First Affiliated Hospital of Dalian Medical University from January 2014 to March 2016 after obtaining informed consent. The investigation project and the informed consent have been approved by the Ethics Committee of the First Affiliated Hospital of Dalian Medical University. The extracted specimens were confirmed to be BC tissue with pathological diagnosis according to the International Union against Cancer (UICC). The specimens were immediately frozen in liquid nitrogen and then stored at −80 °C for analysis.

### Cell culture

BC cells MCF-7 and the MCF-7/ADR were purchased from Nan Jing key GEN Company (Nanjing, China). MCF-7 cells were maintained in 90% DMEM that contained 10% fetal bovine serum (Gibco, Grand Island, NY, USA), 1% penicillin-streptomycin (Gibco, Grand Island, NY, USA). The MCF-7/ADR cell lines were cultured in RPMI 1640 medium (Gibco) with 1 mg/L adriamycin (ADR) to maintain drug resistance. All cells were grown at 37 °C in a humidified atmosphere of 5% CO_2_. Adriamycin was removed from medium 48 h before any experiments were run.

### Real Time PCR analysis

Total RNA including miRNAs was extracted from cell lines using the RNeasy Mini Kit (Qiagen, Valencia, CA) and cDNA was synthesized with QuantiTect Reverse Transcription Kit (QIAGEN, Valencia, CA) according to the manufacturer’s recommended instructions. The primer sequences for qRT-PCR are as follows: hsa-miR-130b mimics, 5′-CAGUGCAAUGAUGAAAGGGCAu-3′; miR-NC, sense: 5′-UUCUCCGAACGUGUCACGUTT-3′; anti-miR-130b, 5′-AUGCCCUUU CAUCAUUGCACUG-3′; anti-miR-NC, 5′-CAGUACUUUUGUGUAGUACAA-3′; siRNA-PTEN, 5′-GGCGUAUACAGGAACAAUATT-3′; and siSCR: 5′-ACTCTAT CTGCACGCTGAC-3′. The qRT-PCR for the analysis of PTEN mRNA expression was performed using the SYBR Green qRT-PCR master mix (TaKaRa, Otsu, Shiga, Japan) and GAPDH (glyceraldehyde-3-phosphate dehydrogenase) as an internal control. The expression level of the target genes was determined relatively to GAPDH and calculated as 2^−(Ct Target gene−Ct GAPDH)^. The expression of miR-130b was measured using mirVana qRT-PCR microRNA Detection Kit according to the manufacturer’s protocol (Ambion Inc., Austin, TX) and normalized by using the 2^−ΔΔCT^ method relative to U6-small nuclear RNA. All the PCR reactions were done in triplicates. The investigation project and the informed consent have been approved by the Ethics Committee of the First Affiliated Hospital of Dalian Medical University.

### Western blot

Total proteins were lysed with lysis buffer supplemented with 1% PMSF, and the concentration was determined using a BCA protein assay kit (Sigma, St Louis, MO, USA). Cell lysates were loaded on 12% polyacrylamide gels and subsequently transferred to a polyvinylidene difluoride membrane. The membranes were blocked with 5% milk and incubated with antibodies against PTEN (1/1,000 diluted; Proteintech, USA), p-Akt 308, p-Akt 473 and Akt (1:1,000 diluted; abgent, San Diego) at 4 °C overnight, followed by incubation with anti-rabbit IgG (1/5000 diluted; GE Healthcare UK Ltd., Little Chalf-ont, UK) at 37 °C for 2 h. GAPDH antibody (1/2500 diluted; Bioworld) was used as an internal standard to normalize protein expression. All bands were detected using ECL Western blot kit (Amersham Biosciences, UK), according to the manufecturer’s protocol.

### Oligonucleotides, plasmids, siRNA and transfection

MiR-130b mimics, negative control oligo-nucleotides (miR-NC), miR-130b inhibitors (anti-miR-130b), negative control oligo-nucleotide (anti-miR-NC), and PTEN pEGFP-N2 vector (PTEN), control vector (vector), small interfering RNA of PTEN (siPTEN), scramble siRNA of PTEN (siSCR) were purchased from RiboBio (Guangzhou, China). MiRNA-130b was overexpressed using miRNA-130b mimics and upregulated PTEN was completed by transfecting with PTEN in MCF-7 cells. In MCF-7/ADR cells miRNA-130b and PTEN were knocked down using a miRNA-130b inhibitor and siPTEN, respectively. Transfection was performed using the Lipofectamine 2000 reagent (Invitrogen) according to the manufacturer’s instruction. The cells were prepared for further analysis 48 h after transfection. The transfection efficiency was evaluated by fluorescence microscopy by calculating the percentage of fluorescein–labelled cells.

### Luciferase assay

A pmirGLO Dual-Luciferase miRNA target expression vector was used for 3′-untranslated region (UTR) luciferase assays (Promega, Madison, WI, USA). Mimics and negative control oligonucleotides for hsa-miR-130b were obtained from RiboBio Co. Ltd. (GenePharma, Shanghai, China). 293 T cells were plated (5 × 10^4^ cells per well) in 24-well plates and cells in each well were co-transfected with hsa-miR-130b mimics and wild type or mutant target sequence using Lipofectamine 2000 (Invitrogen). Cells were then harvested 48 h after transfection and the activities of firefly and Renilla luciferases were measured by using the Dual-Luciferase Reporter Assay System (Promega) and normalized to those of Renilla luciferase activities. The mean of the results from the cells transfected with the miR-control was set at 1.0. Data are presented as the mean value ± SD for triplicate experiments.

### CCK8 assays

The Cell Counting Kit-8 (CCK8) (Dojindo Molecular Technologies) was used to evaluate the viability ability and multidrug chemosensitivity of BC. Briefly, cells (1 × 10^4^) were seeded in 96-well plates (Costar, Charlotte, NC) containing 100 μL full medium, and incubated with or without different anticancer drugs adriamycin (ADR), vincristine (VCR), and paclitaxel (Taxel) (Sigma, St. Louis, MO) for 48 h, respectively. Then cells were treated with 11 μL CCK8. After 4 h incubation at 37 °C in 5% CO_2_, the plates were read under 450 nm wavelengths with a micro plate reader (Bio-Rad Laboratories Inc, Hercules, CA, USA). Each group was carried out in triplicate and repeated three times. The drug resistance was detected by comparing the IC50 values (the drug concentration that induced 50% reduction in cell proliferation) from growth curves.

### Colony Formation assay

The clonogenic survival assay was used to investigate the colony formation ability and chemosensitivity of BC cells exposed to ADR. In short, the cells (1 × 10^3^ cells/well) were plated at in 6-well plates, allowed to attach in 24 h and subsequently exposed to ADR for 24 h. Then, the wells were washed and the culture renewed and the cells incubated for 8 days at 37 °C in a humidified incubator. Finally, the produced colonies were counted after fixing with 10% formaldehyde for 40 min and staining in a 0.1% crystal viola solution for 20 min. The survival curves were plotted using the GraphPad Prism 6 (GraphPad Software, Inc., San Diego, CA, USA).

### Apoptosis assay

Cell apoptosis was determined by FITC Annexin V Apoptosis Detection Kit (BD, Franklin Lakes, NJ, USA). After treatment with chemotherapeutic agent for 48 h, cells were washed twice with cold PBS and then 1 × 10^5^ cells were re-suspended in 100 μL 1X binding buffer, then stained with 5 μL FITC Annexin V and 5 μL PI for 10 min at room temperature (25 °C) in the dark. 400 μL 1X binding buffer was added to each tube. The apoptosis cells were then detected by flow cytometry using fluorescence-activated cell-sorting (FACS) flow cytometer (BD Biosciences, San Jose, CA, USA).

### Immunofluorescence staining

Immunofluorescence staining was performed on cultures after fixing the cells in 10% formaldehyde for 40 min. Then the cells were washed with PBS ten minutes per time, for three times. Non-specific binding was blocked with a 5% BSA phosphate buffer solution for 30 min at room temperature. The cells were then incubated with rabbit polyclonal antibodies specific for Ki67 (diluted at 1: 1000) or PTEN (diluted at 1:100) in 1% BSA blocking buffer overnight at 4 °C. After washing with PBS, the cells were incubated with secondary antibodies (proteintech, USA) diluted at 1:400 in 1% BSA blocking buffer for 30 min at 37 °C in the dark. The cells were washed with PBS and incubated with DAPI (Solarbio) for 5 min at room temperature away from light. Images were obtained on a fluorescence microscope.

### Immunohistochemical staining

The animal tissues were fixed in 4% paraformaldehyde, dehydrated in a graded series of alcohol, and then embedded in paraffin. Slices 0.5 mm thick were cut and dried, deparaffinized, and then endogenous peroxidase was blocked with 0.3% hydrogen peroxide rehydrated for 10 min. After consecutive washing with PBS, the slides were labeled with antibodies PTEN (1:200 diluted, proteintech) and Ki67 (1:1000 diluted, Abcam, Cambridge, UK) at 4 °C overnight. The secondary streptavidin-HRP-conjugated antibody staining (Santa Cruz Biotech, Santa Cruz, CA) was performed at room temperature for 60 min. Finally, the sections were counterstained with hematoxylin and cover-slipped. The Image-ProPlus 4.5 Software (Media Cybernetics) was used to analyze the expression of proteins. Investigation was performed following the approved international guidelines. The investigation project and the informed consent have been approved by the Ethics Committee of the First Affiliated Hospital of Dalian Medical University and the Ethics of Animal Experiments of the Dalian Medical University, China.

### *In vivo* antitumor activity

Animal experiments were performed following the approved international guide for the Care and Use of Laboratory Animals. Four-week-old male athymic nude mice used in the studies were obtained from the Animal Facility of Dalian Medical University, and were provided with sterilized food and water. To establish the BC xenografts, approximately 2 × 10^6^ cells were inoculated subcutaneously into the right flank of each nude mouse, respectively. When mice bearing palpable tumors (about one week after tumor cell inoculation), they were randomly divided into control and treatment groups (n = 6/group). Then, the mice were injected intratumorally with miR-130b mimics or miR-130b inhibitor and their negative controls three times per week for 4 weeks. To identify the chemosensitivity of tumor, the treatment groups received 7 mg/kg adriamycin or normal physiological saline intraperitoneally weekly. Mice were sacrificed and their tumors were isolated, weighed, and photographed. The tumor volume was calculated as follows: Tumor volume = 1/2 (length × width^2^). Tumors were removed, weighed and fixed in 4% paraformaldehyde. The *in vivo* tumorigenesis assays were performed, and growth curves were plotted. These experiments were approved by the Committee on the Ethics of Animal Experiments of the Dalian Medical University, China.

### Statistical analysis

Each experiment was performed at least in triplicate. Data are displayed as mean ± standard deviation (SD) and analyzed by using SPSS 13.0 (SPSS Inc., Chicago, IL). The significance of differences in multiple comparisons was determined using Student’s t test. P < 0.05 was considered to be statistically significant.

## Additional Information

**How to cite this article**: Miao, Y. *et al*. MicroRNA-130b targets PTEN to mediate drug resistance and proliferation of breast cancer cells via the PI3K/Akt signaling pathway. *Sci. Rep.* **7**, 41942; doi: 10.1038/srep41942 (2017).

**Publisher's note:** Springer Nature remains neutral with regard to jurisdictional claims in published maps and institutional affiliations.

## Supplementary Material

Supplementary Information
