# Supplementary material for: MicroRNA-130b targets PTEN to mediate drug resistance and proliferation of breast cancer cells via the PI3K/Akt signaling pathway
Source: Sci Rep. 2017 Feb 6;7:41942. doi: 10.1038/srep41942 (PMC5292739; doi:10.1038/srep41942)
Supplement: Supplementary Information [file srep41942-s1.pdf]

Supplementary Information for

MicroRNA-130b targets PTEN to mediate drug resistance and proliferation of breast cancer cells via the PI3K/Akt signaling pathway

Yuan Miao, Wei Zheng, Nana Li, Zhen Su, Lifen Zhao, Huimin Zhou, Li Jia*

*Correspondence and requests for materials should be addressed to L.J. (jiali0386@sina.com)

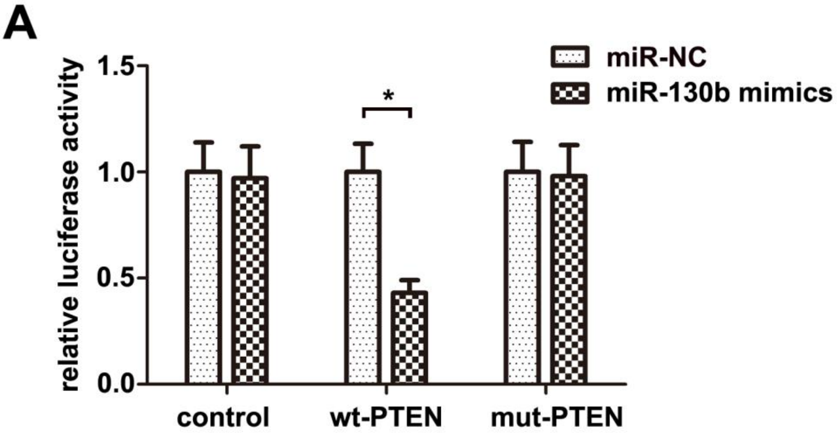

Figure 1.PTEN is a direct target gene of miR-130b. A.The relative luciferase activity of MCF-7cells was detected by luciferase assay after the pGL3-control vector, wt or mut PTEN 3'UTR genes were co-transfected with miR-NC or miR-130b mimics. (*P<0.05). The results are showed as mean ± SD of three independent expriments.
